# Achieving Photonic Spin Hall Effect, Spin-Selective Absorption, and Beam Deflection with a Vanadium Dioxide Metasurface

**DOI:** 10.3390/ma16124259

**Published:** 2023-06-08

**Authors:** Pengfei Zhao, Xinyi Ding, Chuang Li, Shiwei Tang

**Affiliations:** 1 School of Physical Science and Technology, Ningbo University, Ningbo 315211, China; 2 State Key Laboratory of Surface Physics, Department of Physics, Fudan University, Shanghai 200433, China

**Keywords:** tunable metasurface, vanadium dioxide, geometric phase, photonic spin Hall effect

## Abstract

Metasurface-based research with phase-change materials has been a prominent and rapidly developing research field that has drawn considerable attention in recent years. In this paper, we proposed a kind of tunable metasurface based on the simplest metal–insulator–metal structure, which can be realized by the mutual transformation of insulating and metallic states of vanadium dioxide (VO_2_) and can realize the functional switching of photonic spin Hall effect (PSHE), absorption and beam deflection at the same terahertz frequency. When VO_2_ is insulating, combined with the geometric phase, the metasurface can realize PSHE. A normal incident linear polarized wave will be split into two spin-polarized reflection beams traveling in two off-normal directions. When VO_2_ is in the metal state, the designed metasurface can be used as a wave absorber and a deflector, which will completely absorb LCP waves, while the reflected amplitude of RCP waves is 0.828 and deflects. Our design only consists of one layer of artificial structure with two materials and is easy to realize in the experiment compared with the metasurface of a multi-layer structure, which can provide new ideas for the research of tunable multifunctional metasurface.

## 1. Introduction

A metasurface is a kind of ultra-thin two-dimensional plane composed of periodic arrangements of artificial electromagnetic units, whose thickness is much smaller than the working wavelength. It is one of the popular research areas in the field of artificial electromagnetic materials. Compared with traditional metamaterials, metasurfaces have lower loss, thinner thickness, and other advantages. Their unit structure design has high flexibility and tunability, which is easier to process and prepare. After the unremitting exploration of researchers, metasurfaces have shown a wide range of application prospects in many fields, such as metalenses [1,2], waveplates [3], vortex beam generators [4], beam deflection [5], and photonic spin Hall effect (PSHE) [6]. However, most of the current metasurfaces can only achieve a single function, while multifunctional metasurfaces require increasing thickness and complexity, and the function is fixed once determined [7,8]. Therefore, how to integrate multiple functions in one device and dynamically switch functions according to different needs is a very challenging and valuable research direction. This can not only simplify the device structure but also expand the application scope.

In order to achieve the tunability and multifunctionality of metasurfaces [9,10,11,12], many researchers have used tunable materials, such as semi-insulator [13], graphene [14,15,16], and liquid crystal [17,18]. These materials allow metasurfaces to have different optical properties under different conditions, such as tuning of Fano resonance [19], beam steering [17,18], holography [20], and sensing. One of the effective ways to realize switchable metasurfaces is to incorporate standard metasurfaces with phase-change materials (PCMs), such as chalcogenide GeSbTe (GST) alloys [21,22,23] and VO_2_ [24,25,26,27]. However, how to use a single-layer structure of a tunable metasurface to achieve switching between two completely different functionalities is still largely unexplored.

In this paper, we present a novel approach to incorporate VO_2_, a material that exhibits phase transition behavior, into a reflective metasurface with only one artificial structure layer. VO_2_ is a material that responds to temperature changes, and it undergoes a phase transition from a monoclinic insulating phase to a rutile metallic phase at around 68 °C, which leads to significant variations in its conductivity and permittivity. By exploiting this property, we can manipulate the optical functions of the metasurface at different temperatures by applying thermal control. The metasurface that we design is composed of a metal substrate, a dielectric isolation layer, and an artificial structure made of VO_2_ and gold on the top surface. In particular, when VO_2_ is at low temperature, it is in an insulating phase, and the metasurface can generate photonic spin Hall effect, which means that it can split the linearly polarized wave of normal incidence into two circular polarization reflected beams, which propagate in two directions deviating from the normal. When VO_2_ is at a high temperature, it is in a metallic phase, and the metasurface can achieve spin-selective absorption and deflection of circularly polarized light, which means that it can absorb the left-handed polarized light and deflect the right-handed polarized light by about 23.7°. This effect can be used for polarization filtering and switching. The metasurface structure that we propose is very simple and elegant: it only consists of one layer of artificial structure with two materials, which offers an effective route to realize tunable multifunctional metasurfaces.

## 2. The Design of the Tunable Multifunctional Metasurface

**Figure 1:**
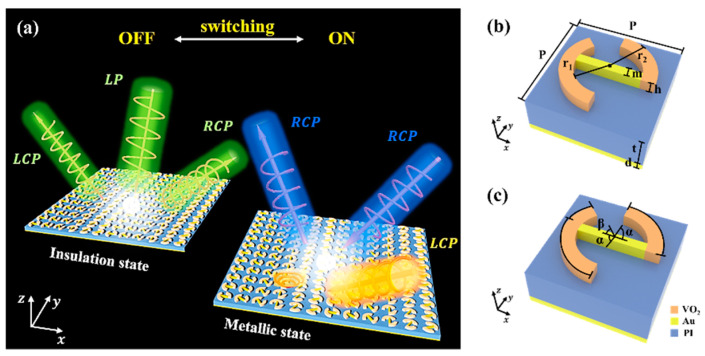
Design principle and structural parameters of the designed switchable multifunctional metasurface. (**a**) The illustration of the switchable multifunctional metasurface; (**b**,**c**) the schematic of the metasurface structural parameters.

As shown in Figure 1, we illustrate the design of the basic unit of our tunable metasurface, which consists of three layers of different materials and structures. The top layer is composed of a phase-change material arc structure and gold strip structure, which are the key elements for achieving phase transition and optical modulation. The middle layer is a polyimide (PI) spacer layer, which serves as a buffer and a thermal insulator between the top and bottom layers. The bottom layer is a gold substrate, which provides a strong reflection and stable support for the whole structure. The geometric parameters of the unit structure are as follows: the period is P=50 μm, the angular sizes of the long arc and short arc structures are α=72° and β=44°, respectively, the radii of the inner and outer arcs are $r_{1}=16\;\mu m$ and $r_{2}=23\;\mu m,$ respectively, the thicknesses of the arc and gold strip structure are both h=4 μm, the thickness of the bottom gold substrate is d=0.5 μm, the thickness of polyimide is t=14 μm, and the width of gold strip structure is m=3 μm.

In this work, we employ VO_2_ as the phase-change material, which can switch between insulating and metallic states under different stimuli, such as temperature, electric field, or light intensity. VO_2_ has a phase transition temperature of around 68 °C. Below this temperature, VO_2_ is in an insulating phase, with a conductivity of σ=200 S/m. Above this temperature, VO_2_ is in a metallic phase, with a conductivity of $\sigma =2\times 10^{5}\;S/m$. The conductivity of VO_2_ can revert to the initial value if the temperature is lowered gradually. Moreover, VO_2_ can also undergo phase transition by other means, such as optical excitation and voltage application, which makes it a very suitable material for our purpose.

We use the finite difference time domain (FDTD) method for numerical simulations in this work. For the unit structure of metasurfaces, we select periodic boundary conditions (PBCs) in the x and y directions and perfectly matched layer (PML) in the z direction as the boundary conditions. For the far-field functions of the metasurface, we select PML as the boundary condition in all three directions: x, y, and z. The dielectric constants of VO_2_ before and after phase transition follow the Drude model:

(1) $$\varepsilon \left(\omega \right)=\varepsilon \prime +\varepsilon \prime \prime =\varepsilon_{\infty }-[\frac{\sigma \omega_{p}^{2}\left(\sigma_{0}\right)}{\sigma_{0}(\omega^{2}+i\gamma \omega )}]$$

where the high-frequency dielectric constant is $\varepsilon_{\infty }=12$, the collision frequency is $\gamma =5.75\times 10^{13}\;rad/s$, $\sigma_{0}=3\times 10^{5}\;S/m$, $\omega_{p}\left(\sigma_{0}\right)=1.4\times 10^{15}\;rad/s$.

The Drude model can also be used to characterize the relative dielectric constant of gold [28]. The parameters of the model are $\varepsilon_{\infty }=1$, the plasma frequency $\omega_{p\_Au}=1.37\times 10^{16}\;rad/s$, and the collision frequency $\gamma_{Au}=1.2\times 10^{14}\;rad/s$.

In this research, we aim to create a tunable metasurface that can switch between different functions by controlling the temperature. VO_2_ can achieve a reversible phase transition from monoclinic to metallic tetragonal structure under specific stimuli, such as temperature change [24,29] and voltage application [26,30,31]. Here, we control the phase transition of VO_2_ by varying the temperature. By crossing the critical temperature of about 68 °C, we can switch between different functionalities. Moreover, in our design, all the structures are on a single layer above the surface, which simplifies the fabrication and manipulation.

## 3. Results and Discussion

### 3.1. VO_2_ Is in the Insulating State

When VO_2_ is in the insulating state at room temperature, it has high reflectivity and low absorption for visible and near-infrared light. In this case, we can design our structure as a Pancharatnam–Berry (PB) phase metasurface, which can realize the PSHE [32]. A PB phase metasurface is a flat optical device that can manipulate the extra phase of incident circularly polarized light by controlling the rotation angle of subwavelength unit structures, thereby achieving various optical functions, such as polarization conversion, beam deflection, holographic imaging, orbital angular momentum generation, and so on [33]. When the linearly polarized light is incident on a PB phase metasurface, it splits into two spin-polarized reflected or transmitted beams, which acquire opposite PB phases due to the geometric phase difference between the unit structures. This leads to the lateral separation of photons with different spin states. This effect originates from the spin–orbit coupling of photons, which describes the interaction between the photon’s spin angular momentum and its orbital angular momentum.

To achieve PB phase metasurface, we first need to design a subwavelength unit structure that can function as a half-wave plate for circularly polarized light. The half-wave plate is a device that can change the polarization state of an incident electromagnetic wave by introducing a phase difference of π between two orthogonal components, such as x- and y-polarization. The structure we designed can function as a half-wave plate because its reflection coefficients for x- and y-polarization have equal magnitudes but opposite phases. By rotating our designed half-wave plate unit structures, we can introduce an additional phase that is twice the rotation angle, which enables us to manipulate any wavefront. By combining the PB phase design with the metasurface, we can achieve a simple structure, a wide working bandwidth, and powerful control over circularly polarized light.

**Figure 2:**
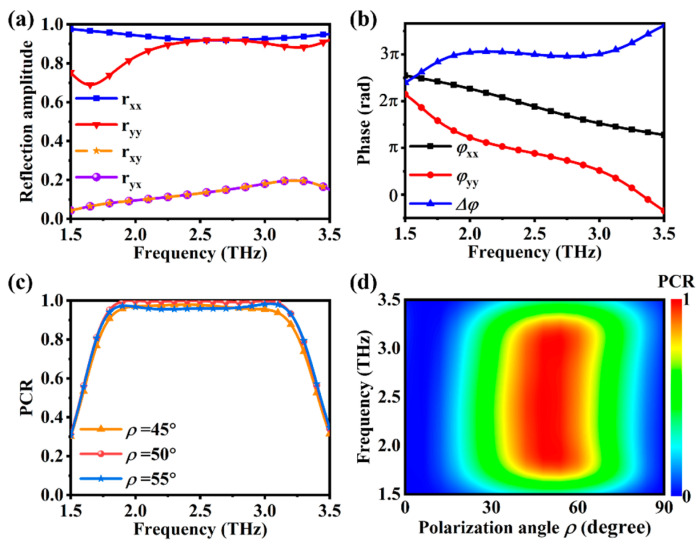
At room temperature, the simulated spectra of (**a**) reflection amplitude and (**b**) phase of the metasurface under the normal incidence of terahertz waves (x- and y- polarizations); (**c**) calculated PCR for polarization angles ρ of 45°, 50°, and 55°; (**d**) calculated PCR for polarization angles ρ from 0° to 90° in the frequency range of 1.5–3.5 THz.

As shown in Figure 2a, in the frequency range of 2.45–2.82 THz, the reflection amplitudes of x- and y-polarizations with normal incidence are almost equal, and the reflection amplitude values are greater than 0.9. Figure 2b indicates that, in the frequency range of 2–3 THz, the phase difference condition $\Delta \varphi =|\varphi_{yy}-\varphi_{xx}|=180{}^{\circ}$ is approximately satisfied.

In order to verify the effect of the half-wave plate, we calculated the polarization conversion rate (PCR) of the structure, which can measure how much the polarization state of the reflected wave deviates from that of the incident wave. The formula for PCR is provided by [34]:

(2) $$PCR=\frac{{\left|r_{yx}\right|}^{2}}{{\left|r_{xx}\right|}^{2}+{\left|r_{yx}\right|}^{2}}$$

where $r_{xx}$, and $r_{yx}$ are the reflection coefficients for different combinations of the incident and reflected polarization.

The polarization angle ρ is the angle between the incident polarization and the x-axis of the metasurface. By adjusting ρ, we can control the polarization state of the reflected wave. The higher the PCR value, the more the reflected wave deviates from the incident wave in polarization. It can be seen from Figure 2c,d that, when ρ is between 40° and 60°, PCR can reach above 0.9. The optimal polarization angle for achieving maximum PCR is ρ=50°, and the PCR can reach above 0.99. It can be seen that the designed half-wave plate is quite consistent with the expected design.

**Figure 3:**
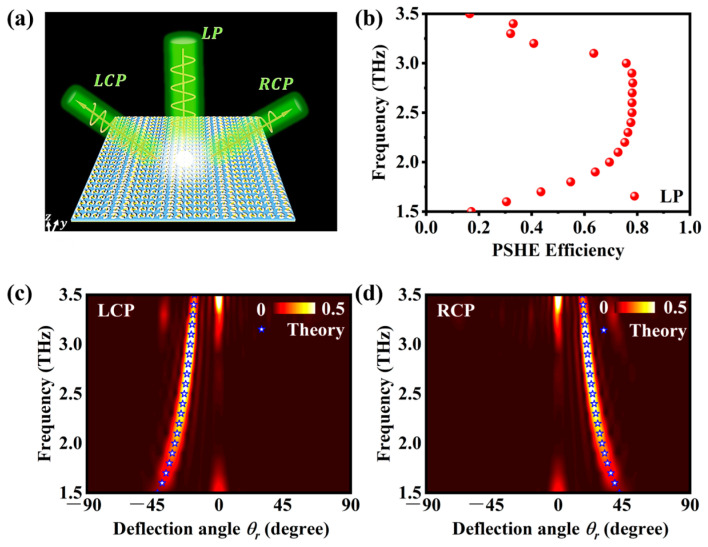
(**a**) The principle of PSHE and the wave plate design. The upper patch of each unit structure has a local orientation η that varies from 0 to π. When the linearly polarized light is incident on the metasurface, it splits into two circularly polarized beams with opposite PB phases; (**b**) the simulated PSHE efficiency as a function of wavelength and incidence angle; (**c**,**d**) the normalized angular distribution of the scattered wave intensity of LCP and RCP at different wavelengths. The deflection angle $\theta_{r}$ of the spin-polarized waves is determined by the phase gradient ξ of the metasurface, which varies with wavelength according to Equation (4).

Based on the principle of the PB phase, we can make the phase shift of the reflected light cover the range of 0–2π by adjusting the local orientation η of the upper patch of the unit structure from 0 to π. By rotating the unit structures, we design a 24 × 24 array structure as a PSHE metasurface, which consists of six unit structures as a supercell. The supercell covers the gradient phase range of 2π by arranging the six unit structures in a phase gradient manner. The supercell is a periodic arrangement of unit structures that forms the basic building block of a metasurface. The principle of PSHE and the wave plate design are shown in Figure 3a. When the linearly polarized light is incident perpendicularly on our designed metasurface, which acts as a half-wave plate, the reflected light becomes two separated LCP and RCP beams.

To calculate the efficiency of PSHE, we use numerical simulation software to simulate the designed wave plate. By integrating the power over the angular region spanned by the reflection mode [35], we can quantitatively estimate the PSHE efficiency, as shown in Figure 3b. According to our simulation, the PSHE efficiency ranges from 69.5% to 78.33% in the frequency range of 2 THz to 3 THz. We also simulated the normalized angular distribution of the scattered wave intensity of LCP and RCP, as shown in Figure 3c,d, where the deflection angle $\theta_{r}$ of the spin-polarized waves can be obtained from the generalized Snell’s law [36]

(3) $$\theta_{r}=\sin^{-1}(\sin\theta_{i}+\xi /k_{0})$$

where $\theta_{i}$ is the incidence angle, $k_{0}=\omega /c$ is the vacuum wave vector and the phase gradient ξ=2π/nP. n is the number of unit structures in a superlattice and P is the period of the unit structure. In our work, we have normal incidence, so we can obtain a simplified formula:

(4) $$\theta_{r}=\sin^{-1}\left(\frac{\lambda }{nP}\right)$$

where λ is the wavelength of the incident light.

From Equation (4), we can see that the deflection angle $\theta_{r}$ of the spin-polarized waves depends on the wavelength of the incident light, and also on the number of meta-atoms n and the period P of the metasurface. When the linearly polarized light is incident at 2.8 THz, according to Equation (4), the deflection angle $\theta_{r}$ of the reflected LCP should be −20.92°, and the actual simulation result is −20.9°, while the deflection angle $\theta_{r}$ of the reflected RCP should be 20.92°, and the actual simulation result is 20.8°. We can see that the simulation results agree well with the theoretical results, and the PSHE efficiency reaches 78.33% at 2.8 THz. From the above analysis, we can conclude that, when VO_2_ is in the insulating state, our designed metasurface can achieve the expected function very well.

### 3.2. VO_2_ Is in the Metallic State

**Figure 4:**
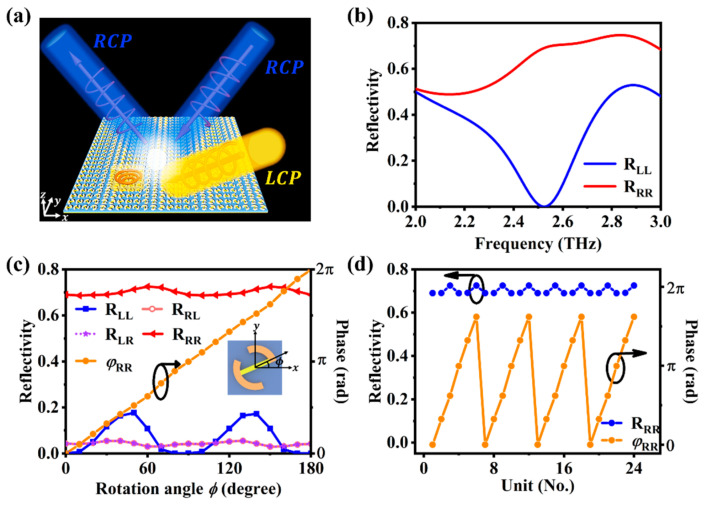
(**a**) Schematic diagram of the metasurface function principle when the temperature is higher than 68 °C; (**b**) reflection amplitude of the incident circularly polarized light; (**c**) reflectance and reflection phase as a function of the rotation angle ϕ; (**d**) calculated reflection phase and reflection amplitude of 24 unit structures when RCP is incident. The circles and arrows indicate whether the data in the figures corresponds to the left or right axis.

Figure 4a shows when VO_2_ reaches the phase transition temperature and is in the metallic state. In this case, the same metasurface design can realize the spin-selective absorption of circularly polarized light and the deflection of the reflected beam. In our design, when LCP light is incident, the metasurface will almost completely absorb the incident LCP light without reflection. However, when RCP light is incident, the reflected wave has high reflectivity and undergoes deflection. This means that the metasurface can act as a spin filter that selectively absorbs or reflects different spin states of light.

We demonstrate the spin-selective absorption of circularly polarized light by simulating the performance of the unit structure, as shown in Figure 4b. At the working frequency of 2.5 THz, when the incident light is RCP, the unit structure reflects 69% of the light with the same polarization ($R_{RR}$), while almost no light is reflected when the incident light is LCP ($R_{LL}\approx 0$). This result confirms that the unit structure can effectively distinguish between different circular polarizations at the working frequency of 2.5 THz, while, for RCP illumination, the main multipole excitation in the unit structure is an electric dipole. However, for LCP illumination, the excitation of antiparallel currents in the nanostructures results in not only the minimization of electric dipole response but also the generation of a pair of parallel magnetic dipoles along the wave propagation direction, which leads to vanishingly small far-field emission [37].

Next, we investigate the dependence of the circularly polarized light reflectance and the reflected RCP phase on the rotation angle ϕ at the operating frequency of 2.5 THz. Figure 4c shows that, when the incident light is LCP, the reflectance is always very low, whereas, when the incident light is RCP, the reflectance of RCP stays almost constant at around 0.7 within the rotation angle ϕ range. Furthermore, we achieve the phase control of optical waves using the geometric phase: a supplementary phase of 2ϕ with reversed spin is added to the circularly polarized optical wave upon reflection by a nanostructure with a rotation angle of ϕ [38]. The phase of the reflected waves can be varied from 0 to 2π by adjusting the rotation angle ϕ from 0 to π, while the amplitudes of the reflected waves remain nearly unchanged.

Therefore, by changing the phase state of VO_2_ from dielectric to metallic, without altering the 24 × 24 array structure that we previously designed in Figure 3, we can still generate a phase gradient along the x direction using the geometric phase, which can deflect the reflected RCP light according to the generalized Snell’s law. As shown in Figure 4d, the phase variation range of the six unit structures that form the supercell covers 2π, which forms a phase gradient along the x direction.

**Figure 5:**
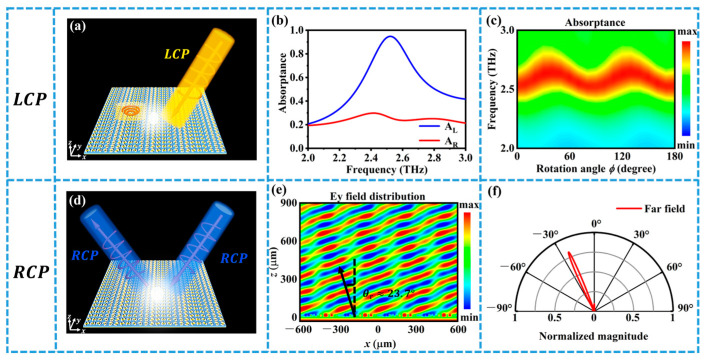
(**a**) Schematic diagram of the metasurface function principle when LCP is incident; (**b**) absorption of the two polarizations of light when circularly polarized light is incident; (**c**) absorption spectra as a function of frequency and rotation angle ϕ; (**d**) schematic diagram of the metasurface function principle when RCP is incident; (**e**,**f**) electric field and far field diagrams when RCP is incident. The direction of the black arrow indicates the direction of the RCP light that is incident vertically and reflected by the metasurface.

Figure 5a illustrates that our tunable metasurface behaves as an absorber when LCP light is incident. This means that the metasurface can convert the incident LCP light into heat and reduce its reflection and transmission. We perform numerical simulations to obtain the absorption rate as a function of frequency. The absorption rate is defined as the ratio of the absorbed power to the incident power. Figure 5b displays the absorption spectra for LCP and RCP incidents separately. The figure reveals that, when LCP light is incident, its absorption reaches a maximum of 0.94 at 2.5 THz, which indicates a high absorption efficiency. To investigate the absorption capability of LCP light as a function of the rotation angle ϕ and frequency, we simulate the absorption of LCP light in the range of 2–3 THz, as shown in Figure 5c. The rotation angle ϕ is the angle between the x-axis and the long axis of the nanorod. The calculation demonstrates that, with the variation in the rotation angle ϕ, LCP light still demonstrates good absorption performance around 2.5 THz, which shows that the absorption effect is robust against the orientation of the metasurface.

Figure 5d shows that our tunable metasurface behaves as a deflector when RCP light is incident. This means that the metasurface can change the direction of the reflected RCP light without changing its polarization state. To investigate the deflection effect, we simulate the electric field distribution and the far field radiation pattern of the reflected RCP light when RCP light is incident, as shown in Figure 5e,f. The figure indicates that, when RCP light is incident, the reflected RCP light deflects to the left by about 23.7°, and the deflection effect is good, with the deflection angle matching well with the theoretical value. The deflection angle can be calculated by Equation (4), which shows that it is proportional to the wavelength of the incident light and inversely proportional to the period of the metasurface. This means that we can tune the deflection angle by changing the wavelength of the incident light or the period of the metasurface. In our work, we also explored whether impurities in VO_2_ have an effect on the performance of the structure [39], and the results show that this effect is actually very small and negligibly affects the performance of our structure.

## 4. Conclusions

We have introduced a novel approach to design a tunable multifunctional metasurface using VO_2_, a phase-change material that can switch between insulating and metallic states under different temperatures. The metasurface consists of three layers: a metal substrate, a dielectric spacer, and an artificial structure on the top surface. The artificial structure is composed of a phase-change material arc structure and gold strip structure, which can manipulate the optical functions of the metasurface at different temperatures. The metasurface can achieve three different functions depending on the phase state of VO_2_: when it is in the insulating state, it can act as a half-wave plate and generate PSHE; when it is in a metallic state, it can achieve spin-selective absorption and deflection of circularly polarized light. The proposed metasurface structure is simple and elegant and offers an effective route to realize tunable multifunctional metasurfaces.

## Data Availability

The authors confirm that the data supporting the findings of this study are available within the article.
